# Comparative assessment of in vitro BBB tight junction integrity following exposure to cigarette smoke and e-cigarette vapor: a quantitative evaluation of the protective effects of metformin using small-molecular-weight paracellular markers

**DOI:** 10.1186/s12987-021-00261-4

**Published:** 2021-06-22

**Authors:** Hossam Kadry, Behnam Noorani, Ulrich Bickel, Thomas J. Abbruscato, Luca Cucullo

**Affiliations:** 1 Department of Pharmaceutical Sciences, Texas Tech University Health Sciences Center, Amarillo, TX 79106 USA; 2 Center for Blood-Brain Barrier Research, Texas Tech University Health Sciences Center, Amarillo, TX 79106 USA; 3 Department of Foundation Medical Studies, Oakland University, William Beaumont School of Medicine586 Pioneer Dr, 460 O’Dowd Hall, Office 415, Rochester, MI 48309 USA

**Keywords:** Tobacco smoke, Electronic cigarettes, JUUL, Permeability markers, Sucrose, BBB, Metformin, Alternative

## Abstract

**Background:**

The blood–brain barrier (BBB) plays a critical role in protecting the central nervous system (CNS) from blood-borne agents and potentially harmful xenobiotics. Our group’s previous data has shown that tobacco smoke (TS) and electronic cigarettes (EC) affect the BBB integrity, increase stroke incidence, and are considered a risk factor for multiple CNS disorders. Metformin was also found to abrogate the adverse effects of TS and EC.

**Methods:**

We used sucrose and mannitol as paracellular markers to quantitatively assess TS and EC’s impact on the BBB in-vitro. Specifically, we used a quantitative platform to determine the harmful effects of smoking on the BBB and study the protective effect of metformin. Using a transwell system and iPSCs-derived BMECs, we assessed TS and EC’s effect on sucrose and mannitol permeability with and without metformin pre-treatment at different time points. Concurrently, using immunofluorescence (IF) and Western blot (WB) techniques, we evaluated the expression and distribution of tight junction proteins, including ZO-1, occludin, and claudin-5.

**Results:**

Our data showed that TS and EC negatively affect sucrose and mannitol permeability starting after 6 h and up to 24 h. The loss of barrier integrity was associated with a reduction of TEER values. While the overall expression level of ZO-1 and occludin was not significantly downregulated, the distribution of ZO-1 was altered, and discontinuation patterns were evident through IF imaging. In contrast to occludin, claudin-5 expression was significantly decreased by TS and EC, as demonstrated by WB and IF data.

**Conclusion:**

In agreement with previous studies, our data showed the metformin could counteract the negative impact of TS and EC on BBB integrity, thus suggesting the possibility of repurposing this drug to afford cerebrovascular protection.

## Background

The blood–brain barrier (BBB) is essential to strictly control and regulate the microenvironment within the central nervous system (CNS) necessary for its normal physiologic function. The BBB plays a vital role in protecting the brain parenchyma from potentially harmful substances, provides a dynamic, effective barrier to the entry of drugs and exogenous compounds into the CNS, and controls the bidirectional transport of biological substances needed to sustain brain metabolism and neuronal function [1]. Therefore it is not surprising that impairments of the BBB have been associated with the onset and/or progression of many neurological disorders [2].

Multiple studies have shown that tobacco smoke (TS) exposure negatively impacts the cerebrovascular system and BBB integrity [3–8], and it is considered a prodromal factor for the onset and progression of many neurological disorders. Growing evidence shows that oxidative stress (OS) plays a critical role in the induction of BBB changes [9, 10]. Indeed, it has been established that TS promotes vascular endothelial dysfunction [4, 6, 11, 12] in a causative and dose-dependent manner [13]. Its harmful effects are strongly related to the smoke’s content of reactive oxygen and nitrogen species (ROS and RNS) [6, 14], nicotine [15–17], inducing oxidative stress-driven inflammation [18]. In addition to its oxidative effects, nicotine has also been reported to affect ions [19] and glucose transporters [20] in cerebral microvessels. Also, chronic subcutaneous infusion of nicotine at a pharmacologically relevant dose produced a significant increase of BBB permeability in-vivo, associated with diminished expression of claudin-3 and altered distribution of ZO-1 mediated by endothelial nicotinic acetylcholine receptors [21].

Over the past decade, various vaping products have hit the market as alternatives to conventional cigarettes and are quickly gaining popularity among adults and adolescents [22]. Electronic nicotine delivery systems or e-cigarettes (EC) have become highly desirable due to the belief that they are much safer than traditional cigarettes [23]. However, similar to TS, chronic vaping could be prodromal to cerebrovascular and neurological impairment [20, 24, 25], although EC’s health impact is not fully understood. Indeed, the limited research and shortage of product guidelines to safely standardize the content of vaping solutions’ have become a critical public and regulatory concern. Various harmful substances such as aldehydes, nitrosamines, acrolein, formic acid, etc., have been detected in the EC vapor [26–29]. Recent studies from our group have shown a significant release of angiogenic, oxidative, and inflammatory factors by BBB endothelial cells in response to tobacco smoke and electronic cigarette vapor extracts (TSe and ECe), which indicate the involvement of common modulators of BBB impairment. The levels of TNF-ɑ, PECAM-1, ICAM-1, and VCAM-1 have significantly increased in a comparable pattern after chronic exposure to tobacco and electronic cigarette smoke [8, 24, 25, 30]. In addition, chronic smoking and vaping have been reported to disrupt the antioxidative response system by downregulating the expression and activity of nuclear factor erythroid 2-related factor 2 (Nrf2). Nrf2 is a crucial modulator of the antioxidant defense response [30, 31]. To this end, metformin has been shown to reduce stress and inhibit the inflammatory responses associated with smoke exposure by upregulating Nrf2 expression [30, 32].

Metformin is an oral medication widely prescribed to manage type 2 diabetes and other metabolic syndromes [33]. It works by inhibiting hepatic gluconeogenesis and increasing peripheral glucose utilization by activating AMP-kinase, which controls blood glucose levels [34]. Beyond its ability to manage diabetes, metformin has been reported to possess anti-inflammatory, anticancer, cardioprotective, hepatoprotective, antioxidant properties. Metformin is currently being investigated as a drug directly acting on the CNS [35–37]. Metformin alleviates chronic inflammation in patients by reducing ICAM-1 and VCAM-1 levels in plasma [38]. Clinically, it has been shown to reduce stroke incidence and diabetes-related death in overweight, diabetic patients [39]. Metformin exerts its action through AMPK dependent and independent pathways [34, 40, 41]. Additionally, several studies have suggested that metformin can be used as a potential candidate for the treatment of different neurodegenerative disorders such as Aging [42], Alzheimer’s disease [43], Parkinson’s disease [44], Multiple Sclerosis [45], and Ischemic Stroke [46]. Having pleiotropic brain effects, metformin is considered a promising candidate to protect against smoke-induced cerebrovascular impairments.

Therefore, the scope of this work was twofold: (1) assess the impact TSe and ECe on BBB integrity in a side-by-side fashion, and (2) assess the protective effect of metformin. The study was carried out using an in vitro BBB model established with iPSC-derived BMECs monolayers cultured on transwell supports. iPSC-derived BMECs allow for the development of a BBB featuring high impedance to the passage of electrical current known as trans-endothelial electrical resistance (TEER), which can be promptly used to assess the barrier integrity where a high TEER value results in low paracellular permeability [47]. This setup is ideal for parallel BBB permeability studies and assesses the barrier's integrity using paracellular markers that are metabolically inert, non-toxic at the applied doses, not bound to other molecules (such as proteins in plasma or tissues), and can be reliably quantifiable [48]. For this purpose, sucrose [^13^C_12_] and mannitol [^13^C_6_] were selected to carry on the permeability studies. We utilized a robust LC–MS/MS method that allows for the simultaneous detection of these tracers with high accuracy and reproducibility [49].

## Methods

### Materials and reagents

Sterile culture wares were purchased from Fisher Scientific (Pittsburgh, PA, USA); reagents and chemicals were purchased from Sigma-Aldrich (St. Louis, MO, USA) or Bio-rad Laboratories (Hercules, CA, USA). Gel electrophoresis was carried out using Mini-Protean^®^ TGXTM gels 4–15% (#456–1084) from Bio-rad Laboratories (Hercules, CA, USA). The antibodies used in this study were obtained from the following sources: Rabbit anti-ZO-1 (#402200) from Invitrogen; mouse anti-Occludin (#331500) from Invitrogen; mouse anti-Claudin 5 (#352500) from Invitrogen. Donkey anti-rabbit (#NA934V) and sheep anti-mouse (#NXA931V) horseradish peroxidase linked secondary antibodies were obtained from GE Healthcare (Piscataway, NJ, USA); goat anti-rabbit (#A32731) conjugated to Alexa Fluor^®^ 488; goat anti-mouse (#A32723, A32727) conjugated to Alexa Fluor^®^ 488 and 555 respectively from Invitrogen (Camarillo, CA, USA).

Small molecular weight markers and their internal standards, [^13^C_6_] mannitol, [^2^H_8_] mannitol, [^13^C_12_] sucrose, and [^2^H_2_] sucrose, were purchased from Omicron Biochemicals (South Hill Street, South Bend, IN, USA). LC–MS/MS grade acetonitrile, water, and analytical grade ammonium hydroxide were obtained from Fisher Scientific (Fair Lawn, NJ, USA). All other chemicals were analytical grade and obtained from commercial sources. Reference full flavor cigarettes (3R4F, 9.4 mg tar, and 0.726 mg nicotine per cigarette) were obtained from the Center for Tobacco Reference Products (Kentucky Tobacco Research & Development Center, Lexington, KY) while e-cigarettes (JUUL pods, Virginia tobacco, 3.0% nicotine strength) were obtained from commercial sources.

### iPSCs differentiation into BMECs

IMR90-c4 induced pluripotent stem cell line was obtained from the WiCell cell repository (WiCell, Madison, WI, USA). iPSCs differentiated into brain microvascular endothelial cells (BMECs) as per the established protocol [50, 51]. Briefly, undifferentiated stem cells were maintained on Matrigel (C-Matrigel; Corning, Corning, MA, USA) coated 6-well plate in Essential 8 medium (E8 Thermo Fisher, Waltham, MA, USA) containing 10 μM Y-27632 (Tocris, Minneapolis, MN, USA) for 3 days before differentiation. Then, differentiation was initiated using an unconditioned medium [UM: Dulbecco’s modified Eagle’s medium/F12 with 15 mM HEPES (Thermo Fisher, Waltham, MA, USA), 20% knockout serum replacement (Thermo Fisher, Waltham, MA, USA), 1% non-essential amino acids (Thermo Fisher, Waltham, MA, USA), 0.5% Glutamax (Thermo Fisher, Waltham, MA, USA) and 0.1 mM β-mercaptoethanol (Sigma-Aldrich, St. Louis, MO, USA)] and maintained for 6 days. On day 6 of differentiation, cells were incubated for 2 more days in EC^++^ media [human serum-free endothelial medium (hESFM, Thermo Fisher, Waltham, MA, USA) supplemented with 1% bovine platelet-poor plasma-derived serum (PDS, Alfa Aesar, Ward Mill, MA, USA), 10 ng/mL bFGF and 10 μM retinoic acid (Sigma- Aldrich)]. On day 8 of differentiation, cells were enzymatically dissociated using Accutase^®^ (Corning) and seeded as single cells on 12-wells transwell systems (polyester, 0.4 μm pore size; filter area 1.1 cm^2^, Corning) pre-coated with a solution of collagen from human placenta (Sigma-Aldrich) and bovine plasma fibronectin (Sigma-Aldrich) (400 μg/ mL collagen IV and 100 μg/mL fibronectin) at a density of 1,000,000 cells/cm^2^ [52]. Please note that higher seeding density is necessary to compensate for the much lower rate of proliferation and higher cell losses of iPSC-BMECs compared to immortalized and primary brain microvascular endothelial cells. 24 h after seeding, media was replaced with EC^−−^ (EC medium supplemented with 1% platelet-poor derived serum). Purified endothelial monolayers were formed on day 10 of the experiment, and barrier integrity tests were performed 48 h after seeding on the transwell system.

### Preparation of soluble tobacco and electronic cigarettes extract

Soluble tobacco smoke (TS) and electronic cigarette (EC) extracts were prepared according to the FTC standard smoking protocol (35 mL draw, 2 s puff duration, 1 puff per 60 s) using a Single Cigarette Smoking Machine (SCSM, CH Technologies Inc., Westwood, NJ, USA) according to previously published methods [7, 24]. TSe and ECe were prepared fresh for each cycle and used in the culture at a 5% dilution (yielding nicotine concentration of approximately 100 ng/mL) [8, 30].

### Treatment of BMECs

After complete differentiation, iPSC-derived BMECs were seeded on 12-well transwell plates for permeability studies, 8-well chamber slides for immunofluorescence imaging, or 6-well plate for western blot analysis (all pre-coated with collagen/fibronectin mixture as previously described). Then, cells were exposed to 5% TSe or ECe for 1, 3, 6, 12, and 24 h. For metformin studies, cells were incubated with 5% extract overnight w/wo concurrent metformin treatment of 10 and 20 μM. These values are within the therapeutic concentration interval that has been reported in patients taking low dose (1500 mg/day) metformin whereas the 20 μM level correspond to the upper limit of this interval [53].

### Cell viability assay

Tetrazolium 3-(4, 5-dimethyl thiazolyl-2)-2, 5-diphenyltetrazolium bromide (MTT) assay was used to assess the cells’ viability [30]. Briefly, following TSe or ECe exposure at 24 h w and wo MF treatment, 5 mg/ml of MTT was added to the cells seeded on 12-well plates and incubated for 3 h at 37 °C. Metabolically active cells converted the yellow MTT to purple formazan crystals. The formazan compound was solubilized using 1 mL of DMSO, and the absorbance was then measured using a Bio-rad plate reader at 570 nm.

### Measurement of barrier function

Barrier integrity of BMECs monolayer was measured by assessing transendothelial electrical resistance (TEER) and paracellular permeability of sucrose and mannitol. TEER measurements were recorded using EVOM STX2 chopstick electrode (World Precision Instruments, Sarasota, FL, USA). The average resistance was obtained by conducting three measurements for each insert (n = 3). Paracellular permeability was assessed by adding 1 mg/mL of [^13^C_6_] mannitol and [^13^C_12_] sucrose to the donor site of the transwell system, followed by collecting 50 μL of aliquots from the acceptor (basolateral chamber) at 10, 20, 30, 60, and 120 min. At the end of the experiment, the donor and acceptor samples were diluted in water in the standard curve range (10–1000 ng/mL). The preparation mentioned above steps were performed to measure the concentrations with LC–MS/MS system. The clearance or permeability-surface area product (PS) for mannitol and sucrose were calculated using the following steps: First, the cleared volume up to each time point was calculated from the following equation:

1 $${\text{Cleared}}\,{\text{volume}} = {\raise0.7ex\hbox{${\left( {C_{{{\text{acceptor}}}} ~ \times ~V_{{{\text{acceptor}}}} } \right)}$} \!\mathord{\left/ {\vphantom {{\left( {C_{{{\text{acceptor}}}} ~ \times ~V_{{{\text{acceptor}}}} } \right)} {C_{{{\text{donor}}}} }}}\right.\kern-\nulldelimiterspace} \!\lower0.7ex\hbox{${C_{{{\text{donor}}}} }$}}$$

here, C_acceptor_ referred to measured concentration in the acceptor compartment at a given sampling time point. V_acceptor_ referred to the acceptor compartment volume, and C_donor_ is the concentration in the donor compartment Eq. (1). Then, linear regression was applied to the plotted cleared volume versus time for samples and blank to obtain the transwell system’s PS. Finally, the permeability coefficient (P) was calculated by the following equations:

2 $$P = {\raise0.7ex\hbox{${PS}$} \!\mathord{\left/ {\vphantom {{PS} S}}\right.\kern-\nulldelimiterspace} \!\lower0.7ex\hbox{$S$}}$$

3 $$\frac{1}{{P_{{{\text{cells}}}} }} = \frac{1}{{P_{{{\text{total}}}} }} - ~\frac{1}{{P_{{{\text{blank}}}} }}$$

The permeability coefficient (P) was obtained by dividing the PS to insert surface area (S) (cm^2^) Eq. (2), and the normalized permeability coefficient of the cell monolayer (P_cells_) was obtained by subtracting the permeability coefficient of the blank wells (P_blank_) from the permeability coefficient of BMECs seeded transwells (Ptotal) Eq. (3).

### LC–MS/MS procedure

Sample preparation steps and LC–MS/MS-based detection of stable isotopes-labeled sucrose and mannitol were performed based on the previously validated method [49]. Briefly, collected samples were diluted in LC–MS/MS grade water in the standard curve range (2–1000 ng/mL). Then, the samples were then subjected to a protein crashing step by diluting at a ratio of 1:9 in acetonitrile: water (80:20) containing 20 ng/mL of [^2^H_8_] mannitol and [^2^H_2_] sucrose followed by centrifugation at 12,000 rpm for 10 min. The supernatant was transported into autosampler inserts and then injected into the LC–MS/MS. ACQUITY BEH amide column (2.1 mm × 50 mm, 1.7 µm; Waters, Milford, MA, USA) was implemented for chromatographic separation using acetonitrile: water: ammonium hydroxide (73:27:0.1, v/v) as the mobile phase, at a flow rate of 0.2 mL/min. The temperature of the column was maintained at 45 °C, and the autosampler was at 4 °C. Electrospray ionization with negative mode was used as the ionization source, and data acquisition and quantification were performed using the Analyst software. The m/z transitions of 353 → 92 and 343 → 71 were used for [^13^C_12_] and [^2^H_2_] sucrose. Also, the transitions of 187 → 92 and 189 → 73 were selected, respectively, to detect [^13^C_6_] and [^2^H_8_] mannitol.

### Preparation of protein extracts and western blot analyses

Cells were lysed using RIPA lysis buffer for cultured cells (Thermo scientific, #78840) as per the manufacturer’s guidelines. Protein concentrations of isolated protein lysates were determined using the bicinchoninic acid assay. Cell lysates containing 40 μg of protein were then subjected to SDS-PAGE gel electrophoresis. Proteins were transferred to polyvinylidene difluoride membrane (PVDF) membranes, blocked in 5% blocking buffer (1% Tween‐20 containing Tris‐buffered saline (TBST) with 5% non‐fat dry milk) to block the non‐specific protein bands for 1 h at room temperature. Membranes were incubated with primary antibody (anti-ZO-1, 1:2000; anti-occludin, 1:500; anti-claudin 5, 1:500) in TBST with 5% non‐fat dry milk at 4 °C overnight. After three times of washing with TBST for 10 min, membranes were incubated with anti‐mouse and anti‐goat IgG‐horseradish peroxidase secondary antibody in TBST with 5% non‐fat dry milk for 2 h at room temperature. Membranes were washed three times, and protein signals were detected by enhanced chemiluminescence‐detecting reagents (Thermo Fisher; Cat#34577) and visualized in X‐ray films in the dark. The protein bands were quantified relative to beta‐actin using Image J software (NIH, Bethesda, MD, USA).

### Immunofluorescence

The iPSC-derived BMECs were seeded in 8-well chamber slides and treated as mentioned earlier. Cells were quickly washed with ice-cold PBS and fixed in 4% paraformaldehyde (PFA, Electron Microscopy Sciences, Hatfield, PA, USA) for 10 min and blocked for 30 min at room temperature (RT) in the presence of PBS supplemented with 10% goat serum (ThermoFisher) and 0.2% Triton-X100 (Sigma). Cells were incubated overnight at 4 °C in primary antibodies targeting claudin-5 (1:100), occludin (1:100), and ZO-1 (1:100) diluted in 10% goat serum (PBSG). After washing three times with PBS, cells were incubated for 1 h at room temperature with Alexa Fluor conjugated secondary antibody in the dark. Finally, cells were washed thrice with PBS and mounted in prolonged gold anti-fade mounting media (Invitrogen, OR, USA). Mounted slides were examined using multi-photon confocal microscopy (Ti-E, Nikon, NY, USA).

### Statistical analysis

The data are presented as mean ± SD. The differences between various treatment groups were analyzed by one-way ANOVA followed by Tukey’s post hoc test (GraphPad Prism, version 8.0, GraphPad Software, La Jolla, CA). An unpaired two-tailed t-test was used for the comparison of the two groups. In all cases, a p < 0.05 was considered significant. All experimental metrics were collected across at least three biological replicates.

## Results

### Evaluation of cell viability

**Fig. 1:**
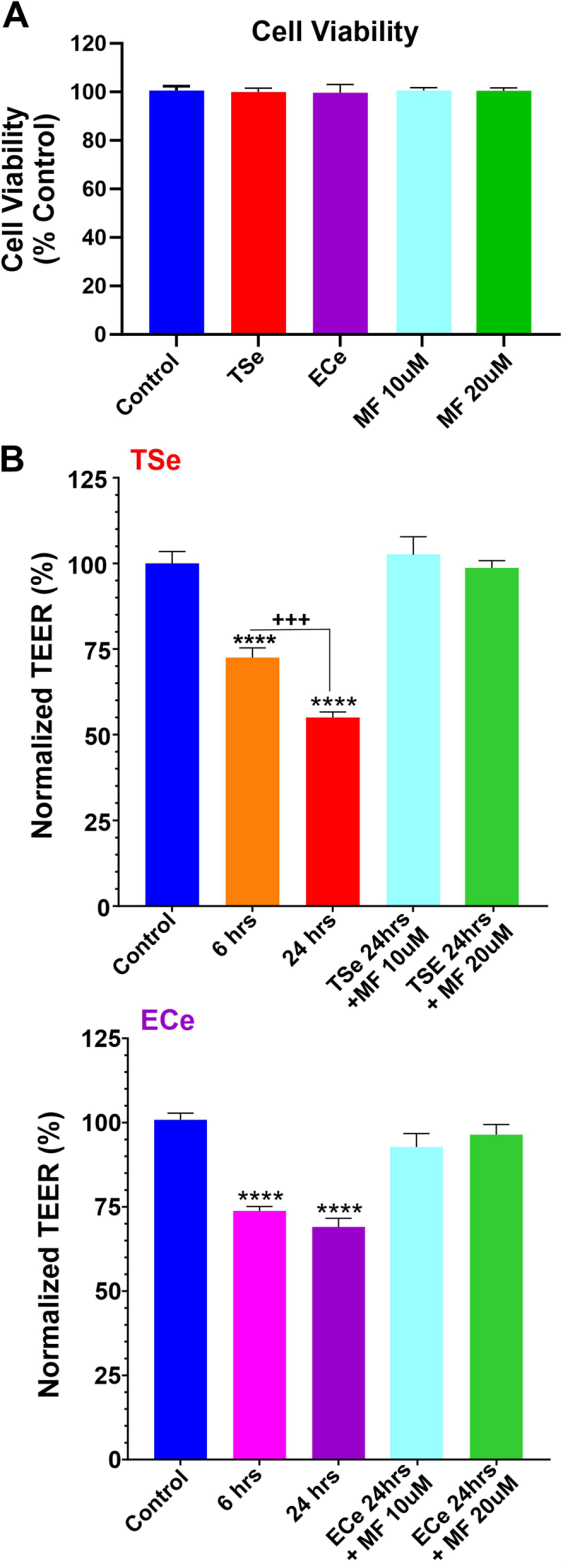
Side-by-side assessment of TS/EC-induced barrier disruption and protective effect of metformin. **A** MTT assay of tested treatments shows no significant impact of TSe, ECe, and MF on cell viability. **B** TSe and ECe negatively impact BBB integrity, as demonstrated by TEER measurement. Pre-treatment with metformin at either 10 or 20 µM concentration abrogated the impact of TSe and ECe on the barrier's integrity. (*p < 0.05; ***p < 0.001; ****p < 0.0001 vs. control; ^**+++**^p < 0.001 vs. 6 h. ANOVA; n = 3 biological replicates)

MTT cytotoxicity assay was performed to evaluate the impact of TSe and ECe 24 h exposure and the MF treatments (10 and 20 µM concentration) on our iPSC-derived BMECs to rule out effect based on cell toxicity and the barrier viability. As shown in Fig. 1A, none of the treatments significantly impacted the cell viability compared to the control.

### Effect of smoke, vapor extracts, and MF on TEER

To assess the effect of TS and EC extracts on BBB integrity, we adopted a transwell system extensively used as an in-vitro model of BBB for drug development and screening [54]. We used iPSC-derived BMECs (which provided high TEER values resulting in low paracellular permeability [47]) to develop a tight endothelial monolayer on Transwell supports to evaluate the BBB's integrity in response to TSe and ECe exposure as well as the protective effect of MF. We used both TEER measurements, sucrose, and mannitol's permeability to assess the integrity and viability of the barrier following each treatment. Initial TEER values, prior testing ranged between 919 ± 18 and 1023 ± 35 Ω cm^2^ for the TSe and ECe setups, respectively which were close to the values reported in the literature [50, 55]. As shown in Fig. 1B, TSE revealed a time-dependent effect impacting the BBB integrity resulting in a 30% decrease of TEER after 6 h (p < 0.001 ANOVA vs. control). The TEER values decreased significantly further (≈ 15% totaling a ≈ 45% from the initial readings at time 0) after 24 h (p < 0.0001 vs. control and p < 0.001 vs. 6 h). Parallel cell cultures treated with ECe (see Fig. 1B) exhibited a similar initial trend resulting in a ≈ 25% drop of TEER within the first 6 h of exposure (p < 0.001). However, the TEER did not drop significantly further when it was re-assessed at 24 h. of exposure. Note also that both MF concentrations tested (10 and 20 µM) effectively protected the integrity of the BBB against TSe or ECe 24 h exposure.

### Effect of smoke extracts on BBB permeability

**Fig. 2:**
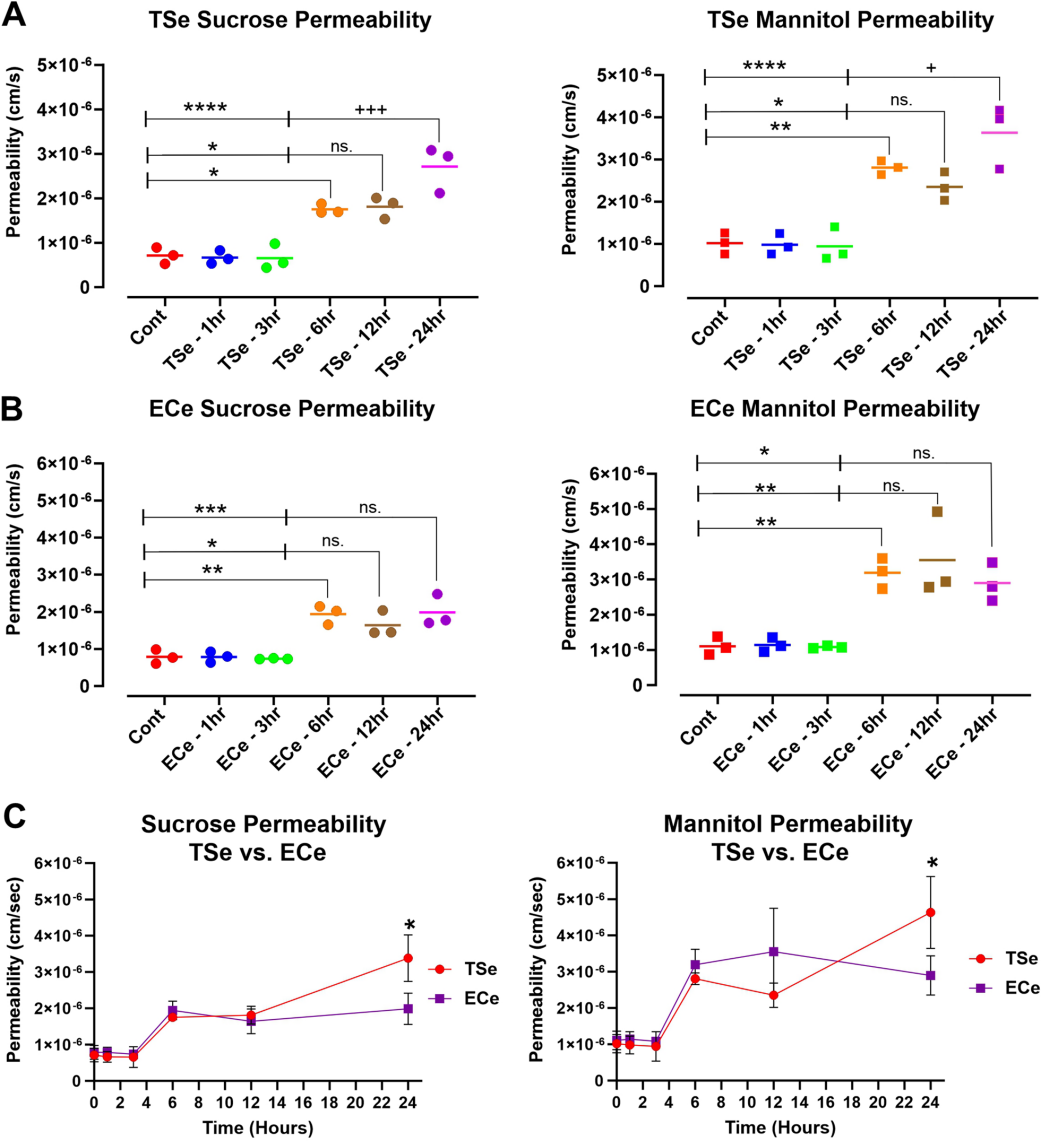
Longitudinal assessment of TSe and ECe impact on BBB permeability. **A** TSe showed a time-dependent increase in both sucrose and mannitol permeability which was significantly evident after 6 h of exposure and further increased at 24 h compared to 6 and 12 h time points. A similar trend was observed in response to ECe treatment (**B**), where increased permeability to either sucrose or mannitol became manifest from the 6 h exposure time point and beyond on. However, permeability measurements at 24 h exposure were not statistically different from those taken at 6 and 12 h. **C** Side by side comparison between TSe and ECe impact on BBB integrity as a sucrose and mannitol permeability function. Note that while the ECe effect plateau after 6 h of exposure, TSe shows a more sustained time-dependent effect which further impairs the integrity of the BBB at 24 h. (*p < 0.05; **p < 0.01; ***p < 0.001; ****p < 0.0001 vs. control; ^**+**^p < 0.05; ^**+++**^p < 0.001 vs. 6 and/or 12 h exposure. ANOVA; n = 3 biological replicates)

We confirmed the monolayer’s barrier function by measuring the in-vitro permeability coefficient of sucrose and mannitol (control values were in the range of 7.11 ± 1.84 × 10^−7^ and 10.2 ± 2.48 × 10^−7^, respectively) through the monolayers. Following the exposure to TSe and ECe at a final 5% concentration for 1, 3, 6, 12, and 24 h, the barrier permeability of these paracellular markers was determined as previously described in the methods section. As shown in Fig. 2A, B, no significant changes were observed after incubation with either TSe or ECe for 1 and 3 h. However, a substantial increase of permeability to either sucrose or mannitol was recorded at 6, 12, and 24 h of exposure. Note also that the impact of TSe exposure at 24 h on the permeability of sucrose and mannitol was significantly worse when compared to either 6 or 12 h time points (p < 0.0001 and p < 0.05 respectively; see Fig. 2A). By contrast, no such difference was observed in parallel cultures exposed to ECe (see Fig. 2B). This is also evident from the longitudinal side-by-side comparison of permeability measurements to sucrose and mannitol in response to TSe and ECe shown in Fig. 2C and reflects the corresponding TEER measurements previously shown in Fig. 1B.

### MF protects the integrity of the BBB against TSe and ECe exposure

**Fig. 3:**
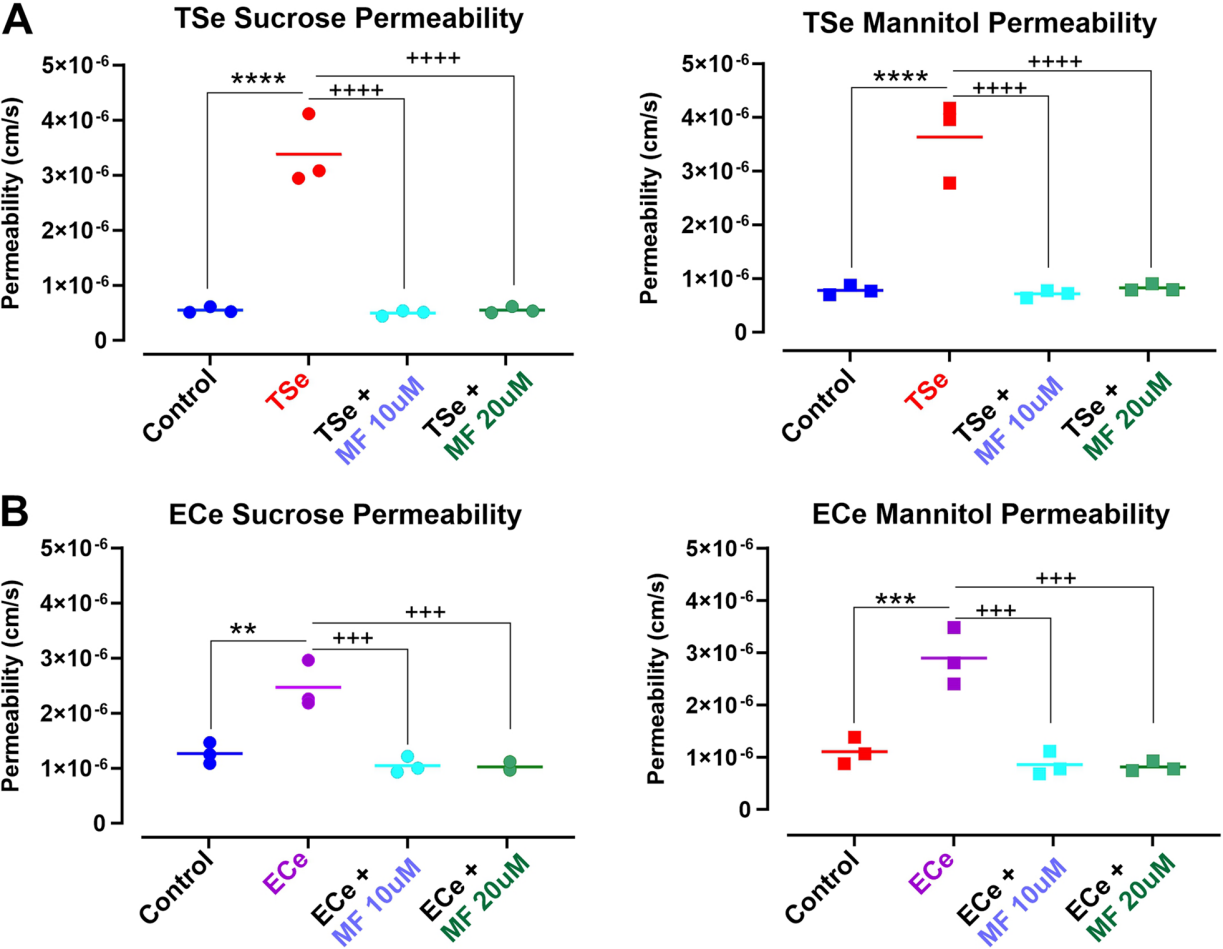
Protective effect of Metformin against **A** TSe and **B** ECe-induced loss of BBB integrity as a function of paracellular permeability to sucrose and mannitol. Treatment with Metformin at two different concentrations (10 and 20 µM) prevented the loss of BBB integrity resulting from the exposure to either TSe (**A**) or ECe (**B**) for 24 h. **p < 0.01; ***p < 0.001; ****p < 0.0001 vs. control; ^**+++**^p < 0.001; ^**++++**^p < 0.0001 vs. TSe or ECe. ANOVA; n = 3 biological replicates)

We tested the protective effects of two concentrations of metformin (10 and 20 μM) comparable to the plasma levels measured in patients [56], which showed lack of toxicity in vitro (see Fig. 1A), while seemingly retaining a BBB protective effect as suggested by the TEER measurements (see Fig. 1B). As shown in Fig. 3, measurement of BBB permeability to sucrose and mannitol at 24 h exposure to either TSe (Fig. 3A) or ECe (Fig. 3B) confirmed impairment of the BBB integrity. This resulted in a significant increase in the permeability coefficient of both sucrose and mannitol. However, co-treatment with MF at either concentration tested prevented the loss of BBB viability. The permeability coefficients of both paracellular markers were not dissimilar from the corresponding controls even after the exposure to TSe or ECe.

### TSe and ECe extracts impact on BBB tight junctions’ and protective effect of MF

To determine whether the negative impact of TS and EC extracts on the BBB integrity was related to alterations of TJs, we assessed the expression and distribution of critical different tight junction proteins using Western blot (WB) and immunofluorescence (IF) assays.

Several conditions are known to increase the permeability of the BBB, including hypoxia and stroke, diabetes, multiple sclerosis, HIV, brain tumors, and peripheral inflammation; changes in the expression and/or organization of TJ proteins have been associated with increased permeability in all of these disease states [57–60].

**Fig. 4:**
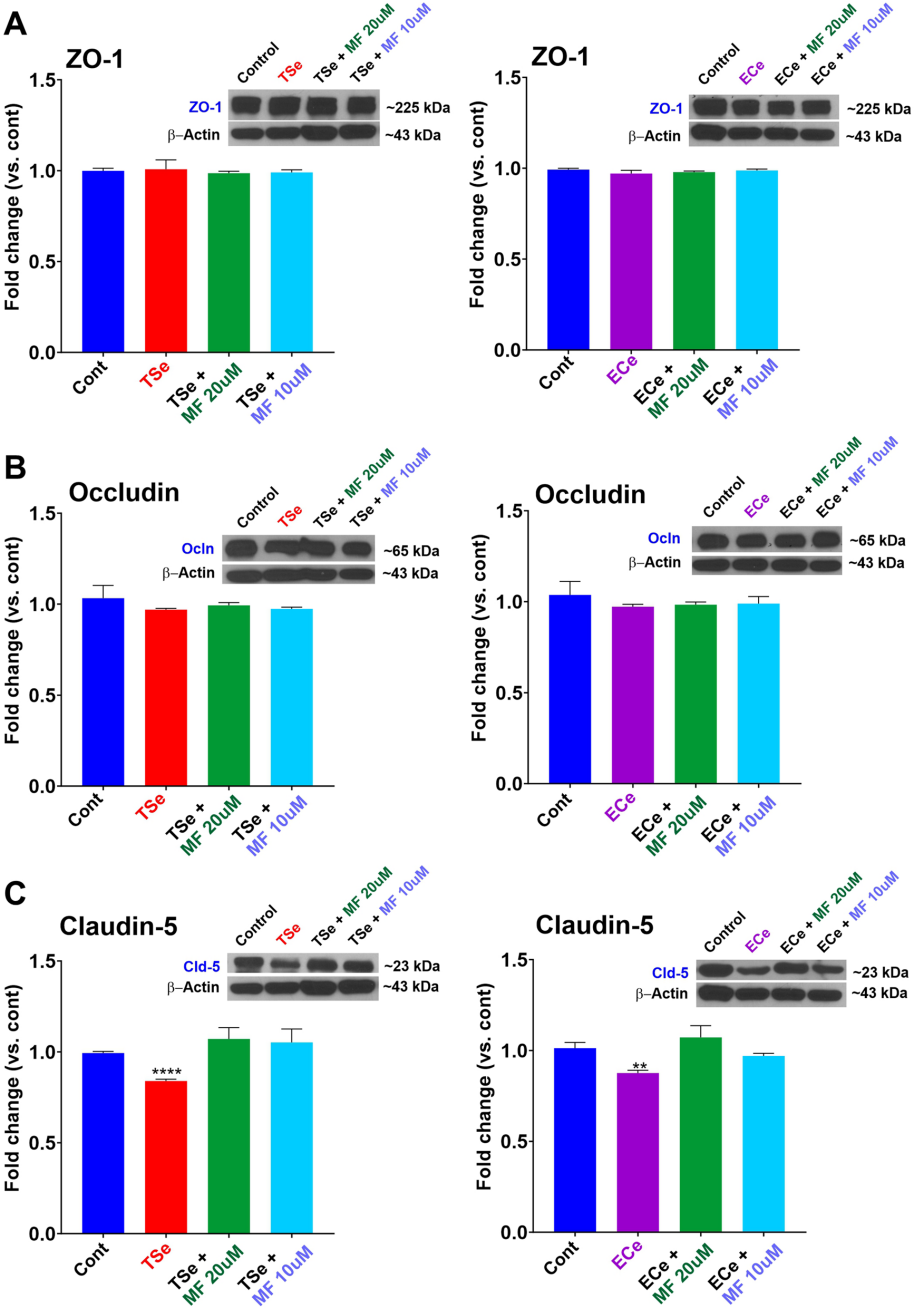
TSe and ECe impact of TJs expression and protective effect of metformin. The expression levels of the TJ proteins ZO-1(**A**) and occludin (**B**) were not significantly affected by exposure to TSe or ECe. However, claudin-5 expression was significantly downregulated by TSe and ECe (**C**). Note also that claudin-5 downregulation by TSe and ECe was abrogated by metformin pre-treatment. WB analyses report protein/β-actin ratios. **p < 0.01; ****p < 0.0001 vs. control. ANOVA; n = 3 biological replicates)

WB analyses of ZO-1 (Fig. 4A1, B1) and occludin (Fig. 4A2, B2) did not reveal any statistically significant change in their expression level in response to TSe or ECe exposure for 24 h. By contrast, the expression level of claudin-5 was significantly downregulated by both TSe and ECe (Fig. 4C1, 2). However, claudin-5 downregulation by TSe and ECe was abrogated by MF pre-treatment at both concentrations tested (10 and 20 µM).

**Fig. 5:**
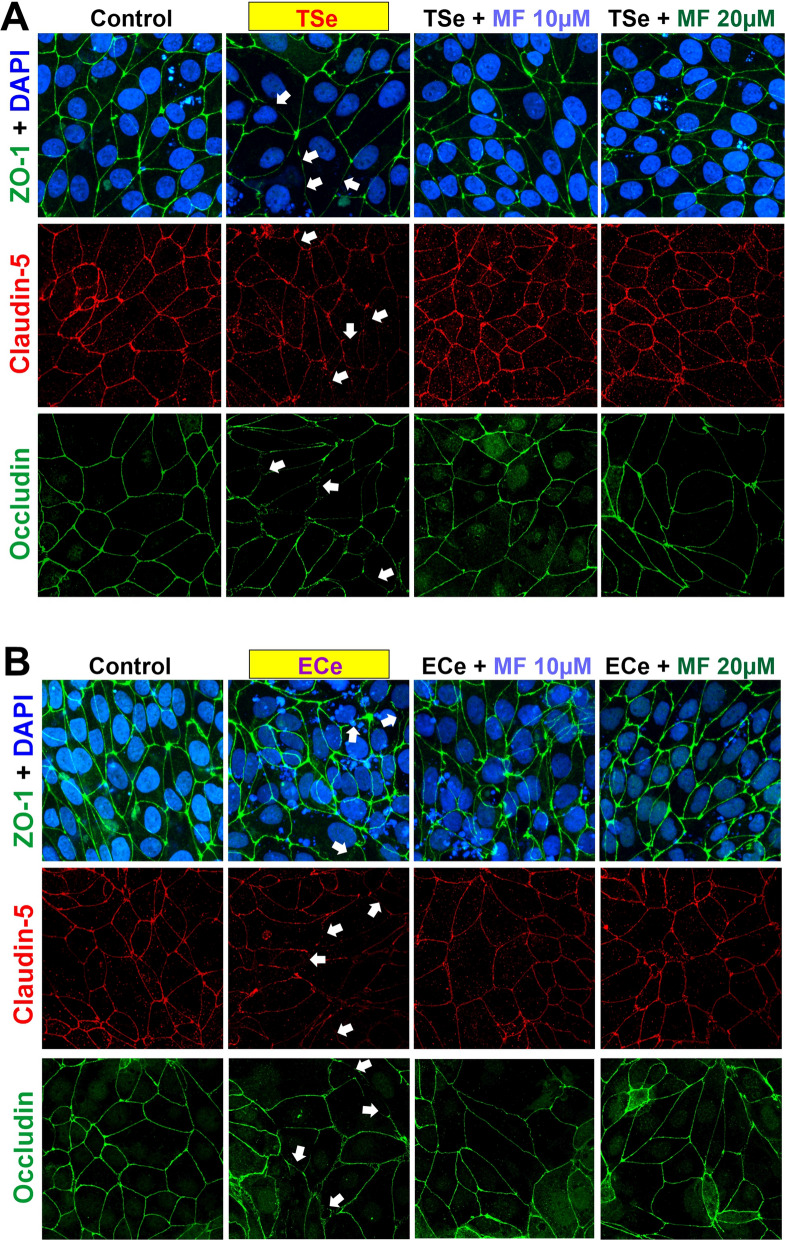
Exposure to TSe and ECe alters the distribution of the TJs, and MF counteracts this effect. Immunofluorescence images show how TSe (**A**) and ECe (**B**) exposure negatively affect the membrane distribution of ZO-1, claudin-5, and occludin. Note the discontinued patterns indicated by *white arrows*. Noticeable is also the reduced expression of claudin-5, as previously shown by WB analyses. Metformin’s protective activity against TSe and ECe effects on TJs distribution and the downregulation of claudin-5 is also evident from the IF images

Analysis of the IF imaging was confirmatory of these results where the expression levels of occludin and ZO-1 were not significantly altered, but that of claudin-5 was markedly decreased (see Fig. 5). However, a discontinuation pattern resulting from the exposure to TSe and ECe was observed for all the TJs examined, including ZO-1, occludin, and claudin-5 (see Fig. 5; white arrows). Pre-treatment with metformin prevented the downregulation of claudin-5 expression and the dysregulation of the TJs distribution, as shown in Fig. 5.

## Discussion

Findings from this in vitro study and previously published work [8, 61, 62] show that TSe and ECe cause dysfunction of the cerebrovascular endothelium. Herein we showed that acute exposure of iPSC-derived BMECs-based BBB monolayers to TS and EC extracts at concentrations that do not induce overt endothelial cell death markedly increased barrier paracellular permeability. We have also shown that TSe and ECe negatively impact the expression level and cellular distribution of the accessory tight junctional proteins ZO-1 and the TJs associated with, including claudin-5 and occludin. In fact, ZO-1 bridges these transmembrane TJ proteins to the actin cytoskeleton [63], thus affecting their ability to form homotypic binding with their corresponding counterparts on adjacent endothelial cells. Alterations of ZO-1 distribution around the cell membrane compromise the endothelial layer and the tightness of paracellular pathways. While TSe and ECe did not affect either ZO-1 or occludin expression levels, claudin-5 was significantly downregulated. This is also noteworthy since claudin-5 has been indicated as the key regulator for the inter endothelial permeability of the BBB [64]. Specifically, claudin 5 plays the selective role of decreasing BBB paracellular permeability of solutes and ions. Structurally, conserved cysteines are crucial for their ability to increase transepithelial resistance, and mutation to any of them eliminates its function [65]. It is also essential to consider that although the expression levels of ZO-1 or occludin were not significantly affected, their distribution pattern was altered. Downregulation of tight junction and dysregulation of their distribution around the BBB endothelium can equally impact the integrity of the BBB. In this specific case the expression level of these TJs does not need to be altered for the BBB to be leaky. In fact, abnormal distribution of the tight can lead to the formation of paracellular gaps between adjacent endothelial cells which compromise the overall integrity of the BBB. Also of note is that in a previous work using *microarray analysis* we have shown that exposure to cigarette smoke also promotes a significant up-regulation of two major ABC efflux transporters, p-glycoprotein (P-gp; ABCB1) and multidrug resistant protein-4 (MRP-4; ABCC4) [7]. It would be interesting in future study to determine if exposure to e-cig vape produces a similar effect and whether this can be abrogated my metformin.

Furthermore, several studies have shown that oxidative stress and inflammation play a critical role in the complex mechanisms underlying BBB disruption. More specifically, ROS production can affect the expression of claudin-5, resulting in increased leakage of solutes and affect the BBB integrity [66, 67]. This is of relevant importance since cigarette smoke is highly enriched with ROS, RNS, NO, and free radicals of organic compounds [14], besides other stable substances that have the potential to increase ROS production [68] or to interact with enzymes that are responsible for ROS generation, such as Nox oxidases [69]. Additionally, nicotine (which is also the primary component of the e-liquid used in electronic cigarettes) can promote cellular oxidative stress on its own [24]. Previous studies have shown that TS and EC extracts elevate ROS levels in primary mBMEC and other cell types, including neuronal, glial, endothelial, and vascular smooth muscle cells [24, 30, 70]. Indeed, exposure to ROS release and/or induced by TS and EC extracts can negatively impact BBB's permeability by affecting ZO-1 protein distribution [71], ultimately resulting in decreased transepithelial electric resistance (TEER) and increased BBB permeability [72].

A common denominator between TS and EC related to oxidative stress damage was identified in the downregulation/deactivation of the nuclear factor erythroid 2-related factor 2 (Nrf2)-are signaling pathway [24, 30] where Nrf2 (a transcription factors regulating the antioxidant defense response) and its downstream signaling molecule NAD(P)H quinone dehydrogenase 1 (NQO-1), promote the activation of a significant number of cytoprotective and detoxification functions [31, 73]. Previous studies by our group have shown that MF can abrogate these TSe/ECe harmful effects by promoting Nrf2 expression/activity, which not only restores the cytoprotective cellular mechanisms but also prevents the loss of BBB integrity, as clearly shown by the TEER measurements and confirmed by the parallel paracellular permeability of sucrose and mannitol (see Figs. 1, 2 and 3) consequent to the downregulation and dysregulation of the BBB’s TJs (see Figs. 4, 5). These results are in line with previous studies from Lie et al. showing that metformin-treated mice exhibited TJs upregulation, including claudin-5 [74], as well as studies from our group, which have shown that TS and EC negatively impacted the BBB integrity leading to decreased TEER values and increased paracellular permeability of FITC (4 kDa) and Rho.BITC (70 kDa) dextrans [24, 25, 30], but the impairment of the BBB was abolished by the concurrent treatment with metformin. Of interest is also the fact that we did not observe any significant dose-dependent difference in terms of drug effects when comparing the 10 µM vs 20 µM dosage. This is in contrast with previous in vivo studies by our group performed in rodent models [30]. A possible explanation is that the concentration differential used in this in vitro study, although in the range interval of metformin serum concentration reported in patients were still reflective of a low dose metformin intake. The dosage used for the in vivo studies where substantially higher (well above the therapeutic concentration used herein) and with a much larger dose interval (100–200 mg/kg daily IP injections corresponding to 77 and 154 µM of MF respectively. In future study we plan to compare MF concentrations reflective of average low and high doses in patients to see if there is an appreciable dose-dependent response to MF in relation to Nrf2 and protection of the BBB while remaining within the therapeutic boundaries (Low–High) of MF.

While the mechanistic link between Nrf2 and TJs expression is unclear and will need to be studied further, these data, along with previously published work by us and others, strongly suggest that metformin could be a viable therapeutic option to offset some of the harmful effects associated with chronic smoking/vaping that can negatively impact the integrity of the BBB and the cerebrovascular system, thus promoting the onset and progression of many neurological disorders [24, 30, 44, 45, 74–76].

## Conclusion

We evaluated the harmful effects of tobacco and electronic cigarettes on BBB integrity. By combining transwells with iPSCs-derived BMECs, the use of small molecular weight paracellular markers, and LC–MS/MS techniques, we precisely quantified TSe and ECe’s impact on BBB paracellular permeability and the effectiveness of metformin as a protective agent. Thus, our data provide a quantitative platform to assess the potential protective efficacy of an agent to counteract the harmful effects of smoking and vaping on the brain microvascular cells. While there is no substitution for quitting smoking and/or vaping, the use of counteractive agents represents a viable alternative for reducing the burden of smoking and vaping on the vast majority of individuals who cannot/will not cease this addictive behavior.

## Data Availability

Not applicable.
